# A Comparative Analysis of PID, Lead, Lag, Lead-Lag, and Cascaded Lead Controllers for a Drug Infusion System

**DOI:** 10.1155/2017/3153252

**Published:** 2017-09-20

**Authors:** Zuwwar Khan Jadoon, Sobia Shakeel, Abeera Saleem, Ali Khaqan, Sana Shuja, Qadeer ul-Hasan, Shahzad A. Malik, Raja Ali Riaz

**Affiliations:** Department of Electrical Engineering, COMSATS Institute of Information Technology, Chak Shahzad Park Road, Islamabad, Pakistan

## Abstract

**Goal:**

The aim of this paper is to conduct a comprehensive comparative analysis between five different controllers for a drug infusion system in total intravenous anesthesia (TIVA) administration.

**Methods:**

The proposed method models a dilution chamber with first order exponential decay characteristics to represent the pharmacokinetic decay of a drug. The dilution chamber is integrated with five different control techniques with a simulation-based comparative analysis performed between them. The design process is conducted using MATLAB SISOTOOL.

**Results:**

The findings show that each controller has its own merits and demerits. The results generated using MATLAB signify and confirm the effectiveness of PI and cascaded lead controllers, with cascaded lead controller as the best control technique to automate and control the propofol delivery.

**Conclusion:**

In this paper, different control techniques for measurement-based feedback-controlled propofol delivery is confirmed with promising results.

**Significance:**

The comparative analysis showed that this drug infusion platform, merged with the proper control technique, will perform eminently in the field of total intravenous anesthesia.

## 1. Introduction

In the field of general anesthesia, target-controlled infusion of anesthesia (TCIA) is a well-recognized technique, found among the most widely recognized closed-loop drug delivery methods [1–3]. Anesthetic drug administration in the human body is a cynosure for the researchers, requiring cardinal attention in the field of biomedical engineering. A paucity or surfeit amount of dose could lead the patient in a precarious condition such as intoxication. Propofol is a nonopioid anesthetic agent, which is widely used for intravenous administration in the field of general anesthesia. Therefore, due to its extensive use and legion advantages, it is exacting to achieve and maintain its target concentration [4, 5].

Pharmacokinetics (PK) is a branch of pharmacology that is related to the study of how a living organism affects the infused drug, while pharmacodynamics (PD) is the branch of pharmacology that is concerned with the study of how the infused drug affects a living organism. Considering all the important parameters, precise pharmacokinetic (PK) and pharmacodynamic (PD) models are developed, on the basis of which TCIA is introduced. The efficacy of TCIA depends on the precision of the PK model, the mutual relation between the effect site concentration and measurement, and the device used for drug monitoring and control. To achieve radical control over the drug delivery, continuous propofol measurement is performed throughout total intravenous anesthesia (TIVA).

In [6, 7], a canonical three-compartmental model has been used to describe the qualities and peculiarities of drug concentration and dynamical mobility inside the human circulatory system. The first compartment constitutes the volume of blood, where drug is injected. After the drug delivery, the drug concentration in different individual compartments including the effect site, that is, the brain, is impacted due to various factors. These factors include distribution rate constants within interacting compartments, volume comprising the respective compartments and rate of the drug infusion.

Setting the drug concentration degree at the effect site to the desired targeted degree is precisely the main goal of TCIA. The drug is infused and measured in the blood that is the first compartment. In such case, by using the pharmacodynamic models, measurement of drug concentration in the blood provides an estimation of effect site drug concentration. Once the drug concentration is quantified in the blood, its control and regulation to a desired targeted level can also be accomplished.

In a closed-loop drug delivery system, different control techniques are employed in order to control the drug infusion rate, which leads to the control of drug concentration in the blood and therefore at the effect site. In [8–11], PID controllers are discussed as being the simplest controllers, which are widely used in the field of control system design. References [12, 13] discuss vulnerability to high-frequency noise and integrator windup effect as one of the main disadvantages of a PID controller. The most viable alternatives are lead, lag, or an adjustment of both controllers, where design parameters are available, studied in [14, 15]. In some cases, discussed in [16, 17], second order lead and lag controllers, compared with the widely used PID controllers, lead to better results.

The phase lead and phase lag are two of the most commonly utilized control architectures for designing control systems with the root locus or bode compensation approach used. In [18], the cascade or series compensation is stated as the most popular method in the control system design. The design and implementation of lead and lag controllers have been studied in [19–22]. A conceptual understanding in control system engineering is provided in [23]. A comparative analysis of different control techniques is studied in [24, 25].

This paper focuses on the comparative analysis of five different control techniques and their design process, to automate the delivery of an infusion system, based on propofol measurement in a closed-loop feedback system. The comparative analysis is conducted with respect to different time domain specifications like gain, percentage overshoot, settling time, and rise time. The design process of phase-lead, lag, lead-lag, and cascaded lead controllers is performed by applying the principles of the root locus technique [26-28], using MATLAB SISOTOOL [29-31]. The simulation study is based on the model developed by Myers et al. in [32].

This paper is organized as follows: Section 2 describes the system modeling and its description.  Section 3 presents the methods employed in an open-loop and closed-loop analysis.  Section 4 shows the simulation results for five different controllers, and the comparative analysis has been drawn on the basis of different parameters for example rise time, overshoot, and settling time.  Section 5 comments on the discussion of the paper. Finally, Section 6 concludes the paper.

## 2. System Model and Description

The following equations, as shown by Myers et al. in [32], are used in system modeling.

After the introduction of a bolus injection or propofol infusion, a first-order kinetics model depicts the drug elimination dynamics in blood circulation, mathematically described as

(1) $$\begin{array}{c} \frac{d\mathbb{C}\left(t\right)}{dt}=-\lambda \mathbb{C}\left(t\right). \end{array}$$

The model represents an exponential decay in drug concentration over time

(2) $$\begin{array}{c} \mathbb{C}\left(t\right)=\mathbb{C}\left(t_{0}\right)e^{-\lambda t}, \end{array}$$

where ℂ(*t*) represents the time-dependent drug concentration and ℂ(*t*_0_) describes the concentration of drug at time, *t* = *t*_0_. *λ* represents the drug elimination rate which is a function of the volume of the blood and PK decay rate.

When the drug is injected in the blood at an infusion rate *i*(*t*), (1) is qualified as

(3) $$\begin{array}{c} \frac{d\mathbb{C}\left(t\right)}{dt}=-\lambda \mathbb{C}\left(t\right)+\frac{i\left(t\right)}{V}. \end{array}$$

In our simulation study, a dilution chamber of constant volume is used to mimic the human blood. The exponential decay of drug concentration in this chamber models the actual PK decay of the drug in the blood. The drug elimination rate *λ* is determined as

(4) $$\begin{array}{c} \lambda =\frac{\Omega }{V_{1}}. \end{array}$$

*𝕍*_1_ shows the volume of the chamber and *Ω* represents the dilution flow rate.

A particular amount of drug injection increases the concentration level inside the chamber. This new concentration of the chamber is defined as initial concentration, that is, ℂ_1_(0), which decays exponentially over time. The decaying exponential rate is set by *Ω* and *𝕍*_1_

(5) $$\begin{array}{c} {\mathbb{C}}_{1}\left(t\right)={\mathbb{C}}_{1}\left(0\right)e^{-\left(\Omega /V_{1}\right)t}. \end{array}$$

The dynamics of concentration in the dilution chamber can equally be symbolized in the derivative form as

(6) $$\begin{array}{c} \frac{d{\mathbb{C}}_{1}\left(t\right)}{dt}=-\frac{\Omega }{V_{1}}{\mathbb{C}}_{1}\left(t\right). \end{array}$$

The bolus dose, when merged with continuous infusion results in the time-dependent concentration, changes in the dilution chamber as

(7) $$\begin{array}{c} \frac{d{\mathbb{C}}_{1}\left(t\right)}{dt}=-\frac{\Omega }{V_{1}}{\mathbb{C}}_{1}\left(t\right)+\frac{i\left(t\right)}{V_{1}}. \end{array}$$

The system model in terms of its differential equation is represented by (7).

**Figure 1:**
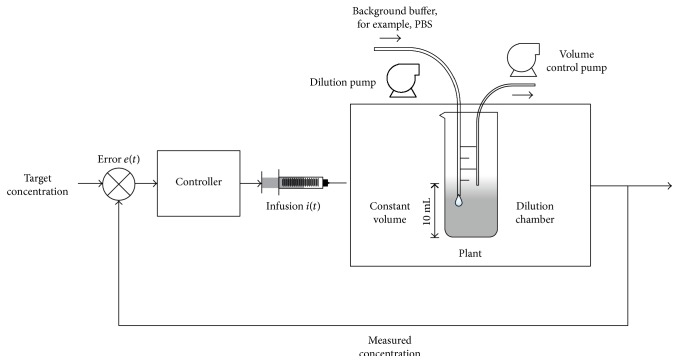
Block diagram of a feedback-controlled drug infusion system.

Figure 1 shows the block diagram of the feedback-controlled drug infusion system. It is comprised of the following:

1. A controller to achieve the target concentration of propofol
2. A constant volume dilution chamber, representing the first compartment, which is our plant
3. A dilution pump to regulate the continuous dilution rate of the background electrolyte
4. A volume control pump to maintain a constant volume in the chamber.

## 3. Methods

To analyze the performance of the feedback-controlled drug infusion system, a dilution chamber with constant volume *𝕍*_1_ is considered. In order to vary the initial concentration ℂ_1_(0) over time, the dilution chamber is diluted continuously by a background electrolyte via a dilution pump at a dilution rate (*Ω*). The concentration change from an initial concentration inside the dilution chamber is described in (3). At first, the system is analyzed in an open loop, following the closed-loop system design and implementation, using five different control schemes.

### 3.1. Open-Loop Analysis

The open-loop analysis can be described by the following two steps. The first step describes the exponential decay of the dilution chamber. *𝕍*_1_ describing the volume of the chamber is fixed at 10 mL. ℂ_1_(0) describing the initial concentration of the propofol was set to 0.01 mL. In order to the mimic the PK decay, this initial concentration is diluted by the continuous dilution of a background electrolyte at a flow rate of 0.04 mL/s.

In the second step, the same volume is maintained in the dilution chamber, that is, 10 mL; however, the concentration of propofol in the chamber is set to zero, that is, ℂ_1_(0) = 0. Afterwards, both continuous dilution and propofol infusion are processed in a parallel operation with a fixed infusion rate.

**Figure 2:**
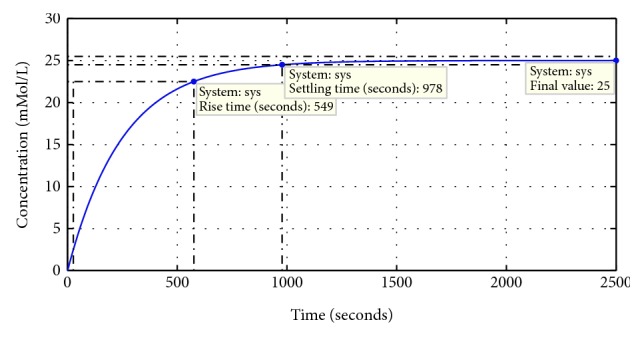
Step response of the uncontrolled dilution chamber.

The design parameters of the open-loop analysis are shown in Table 1.  Figure 2 shows the open-loop step response of the uncontrolled system with a very high settling time, rise time, and steady state error. However, the overshoot percentage in the system response is almost zero.

### 3.2. Control Techniques and Designs

This section presents the closed-loop design analysis of the drug infusion system. In order to achieve the target-controlled drug infusion in the first compartment or the dilution chamber, five different control techniques are designed and implemented in a closed-loop feedback system. The fundamentals of the techniques employed in the meticulous design process of all five dynamic controllers are referred to [33–35]. The design techniques described in the closed-loop analysis of the infusion system uses the same parameters as have been used in the open-loop analysis. The design parameters are shown in Table 1.

#### 3.2.1. PID Controller

The proportional-integral-derivative (PID) controller is one of the most commonly used and universally accepted control algorithm used in control industry. The PID controller is popular for its attributes partly to their wide-range operation of robust performance and partly to their simplified operation. The term PID describes its three main components, a proportional control term (*K_P_*), an integral control term (*K_I_*), and a differential control term (*K_D_*).  Table 2 describes the effect of a PID controller in terms of its three components. Mathematically, the following equations describe a PID controller:

(8) $$\begin{array}{c} e\left(t\right)=target-\mathbb{C}\left(t\right), \end{array}$$

(9) $$\begin{array}{c} i\left(t\right)=K_{P}e\left(t\right)+K_{I}\int_{0}^{t}e\left(t\right)dt+K_{D}\frac{de\left(t\right)}{dt}. \end{array}$$

By keeping the same parameters, as used in the open-loop analysis, a PID controller is designed to yield the targeted concentration. In the study, conducted by Myers et al. in [32], a PID controller is triggered after the bolus injection is injected. A minor amount of fluctuation in drug concentration is observed. Therefore, *K_D_* part of the controller is set to zero. By connecting the PI controller with the dilution chamber in a closed-loop system, nine runs are recorded for each of the nine parameters in Table 3. Finally, the PI controller is tuned by using parameter 5 to control the closed-loop drug delivery. The parameter set employed in our simulation study is identified from the experimental and simulation parameters in [32].

**Figure 3:**
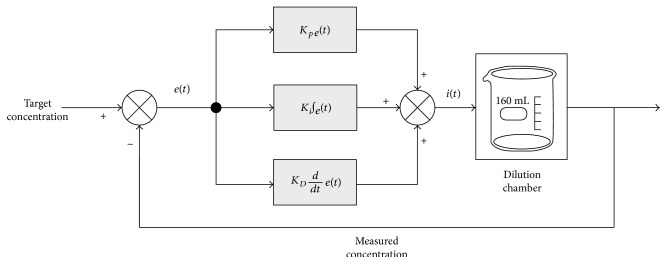
Block diagram of a PID controller connected with the dilution chamber in a closed-loop feedback system.

The open-loop dynamics plus the system modeling represented in (7) and the mathematical description of the PID controller in (8) and (9) collectively present a complete picture of the closed-loop flow system. A PID controller, incorporated with the dilution chamber in a closed-loop feedback system, is illustrated in Figure 3. By tuning the PI controller using parameter 5, that is, *K_P_* = 4.5 × 10^6^ and *K_I_* = 55, the closed-loop transfer function of the PI-controlled dilution chamber is obtained as

(10) $$\begin{array}{c} G_{CL}\left(s\right)=\frac{4,500,000s+55}{10s^{2}+4,500,000s+55}. \end{array}$$

#### 3.2.2. Phase-Lag Controller

By adding equal numbers of poles and zeros, a phase-lag controller provides an appreciable amount of relative stability to a system, yielding slow response time. In a phase-lag controller, the pole of the controller is placed closer to the origin as compared to the zero of the controller. The generalized transfer function of a phase-lag controller is given as

(11) $$\begin{array}{c} C\left(s\right)=\frac{s+z}{s+p}, \end{array}$$

where

(12) $$\begin{array}{c} p<z. \end{array}$$

To address the steady state error, a phase-lag controller is designed, using the root locus method. The target is to achieve a compensated steady state error value of *E*_ssc_ *<* 0.01, without affecting the transients, where *E*_ssc_ represents the compensated steady state error. In case of a lag controller, varying the transients leads to slower response time [35], which is not desired. The phase-lag controller is designed using the following two steps.

In the first step, the *z*/*p* ratio is evaluated. Suppose we have a system *G*(*s*), incorporated with a lag controller *C*(*s*) = (*s* − *z*)/(*s* − *p*).

Using the final value theorem [36], first, a formula for steady state error (final value of the error) is calculated

(13) $$\begin{array}{c} E_{ss}=\underset{s\to 0}{\text{lim}}s\cdot \frac{U\left(s\right)}{1+\left(G_{N}\left(s\right)/G_{D}\left(s\right)\right)\cdot \left(\left(s-z\right)/\left(s-p\right)\right)}. \end{array}$$

For step input, *U*(*s*) = 1/*s*, the above equation, after applying the limit, simplifies to

(14) $$\begin{array}{c} E_{ss}=\frac{G_{D}\left(0\right)\cdot p}{G_{D}\left(0\right)\cdot p+G_{N}\left(0\right)\cdot z}. \end{array}$$

Now, by solving for the ratio of the zero to the pole, we get the following final equation:

(15) $$\begin{array}{c} \frac{z}{p}=\frac{G_{D}\left(0\right)-E_{ssc}G_{D}\left(0\right)}{E_{ssc}G_{N}\left(0\right)}, \end{array}$$

where *z* and *p* represent the zero and pole of the controller, while *G_D_* (0) and *G_N_* (0) are the denominator and numerator of the system transfer function *G*(*s*) at *s* → 0.

**Figure 4:**
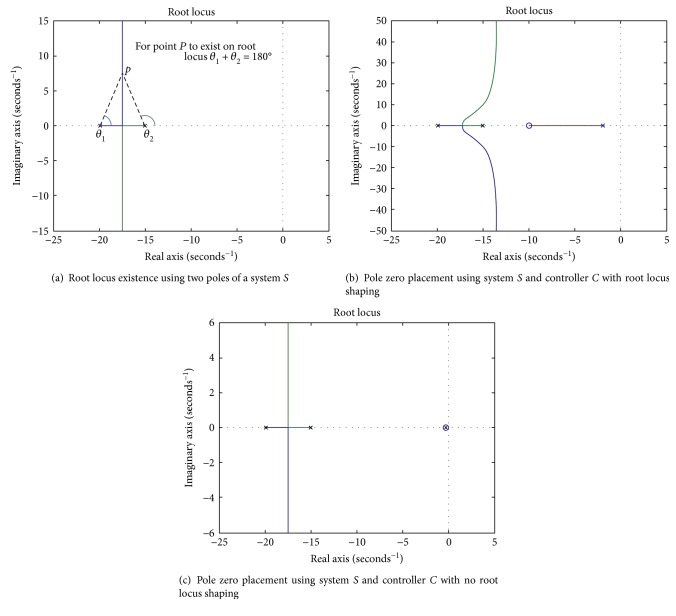
Graphical illustration of root locus existence shaping and the concept of pole zero placement using a pseudo system *S* and a pseudo lag controller *C*.

In the second step, the location of the zero and pole of the controller is determined using an algebraic approach. In order for a point *P* to exist on the root locus, the sum of the angles of all the poles minus the sum of the angles of all the zeros has to equal 180° [36]. The angle stated corresponds to the angle of the line from the open-loop pole or zero to that from point *P* on the root locus.  Figure 4(a) graphically explains the above concept of root locus existence by considering a second-order pseudo system *S*.

Mathematically,

(16) $$\begin{array}{c} \sum \theta_{p}-\sum \theta_{z}=180^{{}^{\circ}}. \end{array}$$

Now, to delineate the pole zero placement (design process) of the *actual* phase-lag controller, the same second-order pseudo system *S* is considered and incorporated with a pseudo phase-lag controller *C*.  Figure 4(b) shows the changed root locus and a considerable amount of root locus shaping of the system *S*, when merged with controller *C*. Following the concept of the root locus existence from (16),

(17) $$\begin{array}{c} \theta_{1}+\theta_{2}+\theta_{p}-\theta_{z}=180^{{}^{\circ}}, \end{array}$$

where *θ*_1_ and *θ*_2_ are the angles associated with the poles of the pseudo system *S* and *θ_p_* and *θ_z_* are the angles associated with the pole and zero of the pseudo lag controller *C*. Now, in order to have no root locus shaping, the angle contribution of both the pole and zero of the controller *C* should be close to zero, as shown in Figure 4(c) Mathematically,

(18) $$\begin{array}{c} \theta_{p}-\theta_{z}\approx 0. \end{array}$$

Now, considering the *actual* system, that is, the dilution chamber, to achieve the above two requirements of negligible angle difference and minimum root locus shaping as shown in (18) and Figure 4(c), respectively, the following procedure is implemented. The pole and zero of the actual phase-lag controller are placed closer to each other and to the origin with the same *z*/*p* ratio as calculated in the first step using (15). By doing such placement, the angles for the pole and zero became closer and closer together and the requirement in (18) is met. Pole zero placement of the controller is found using (19) and (20). The gain (*k*), required to achieve the desired steady state error, is computed using MATLAB SISOTOOL.

(19) $$\begin{array}{c} z=\frac{real\;closed‐loop\;dominant\;pole}{50}, \end{array}$$

(20) $$\begin{array}{c} p=\frac{z}{z/p}. \end{array}$$

**Figure 5:**
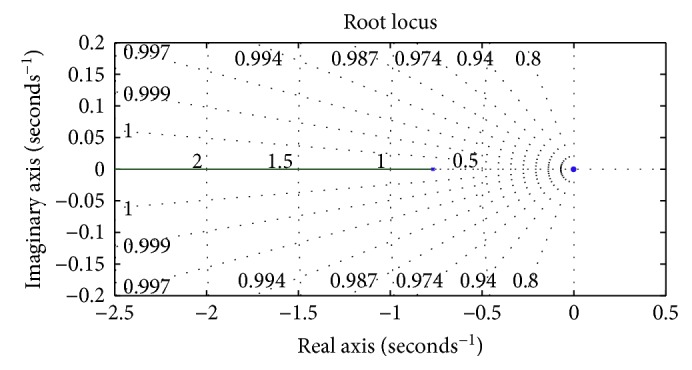
Root locus of the system incorporated with the designed lag controller.

The appropriate placement and zero-angle contribution (*θ*_*p*_ − *θ*_*z*_ ≈ 0) of the pole and zero of the controller is shown in Figure 5, verifying no root locus shaping. The bottom line of the steps involved in the whole designing process is stated as the following:

1. The *z*/*p* ratio is maintained, which is needed for the compensated steady state error (*E*_ssc_).
2. The *z* and *p* of the lag controller are kept as close to the imaginary axis as possible to avoid shaping the root locus and the transients.

**Figure 6:**
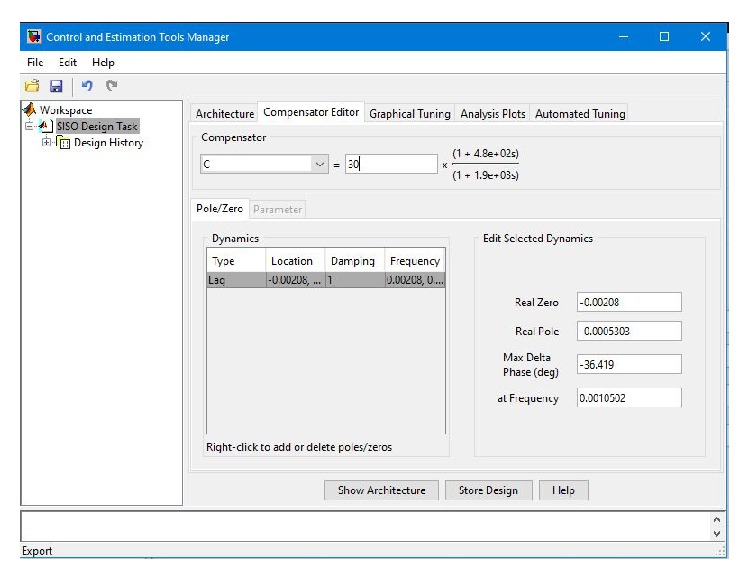
Illustration of the design details of the phase-lag controller.

The design details of the phase-lag controller are illustrated in Figure 6. The transfer function of the designed phase-lag controller with adjusted gain *k* = 30 is

(21) $$\begin{array}{c} G_{c}\left(s\right)=\frac{7.5757\left(s+0.0021\right)}{s+0.0005303}. \end{array}$$

The controller, when merged with the dilution chamber, resulted in the following closed-loop transfer function

(22) $$\begin{array}{c} G_{CL}\left(s\right)=\frac{0.75757\left(s+0.0021\right)}{\left(s+0.002096\right)\left(s+0.76\right)}. \end{array}$$

#### 3.2.3. Phase-Lead Controller

By adding equal numbers of poles and zeros, a phase-lead controller provides an appreciable improvement in the transient response of a system, increasing the open-loop gain in some cases. Increasing gain leads to more susceptibility to noise. In case of a lead controller, the zero of the controller is placed closer to the origin as compared to the pole of the controller. The generalized transfer function of a phase-lead controller is given as

(23) $$\begin{array}{c} C\left(s\right)=\frac{s+z}{s+p}, \end{array}$$

where

(24) $$\begin{array}{c} p>z. \end{array}$$

**Figure 7:**
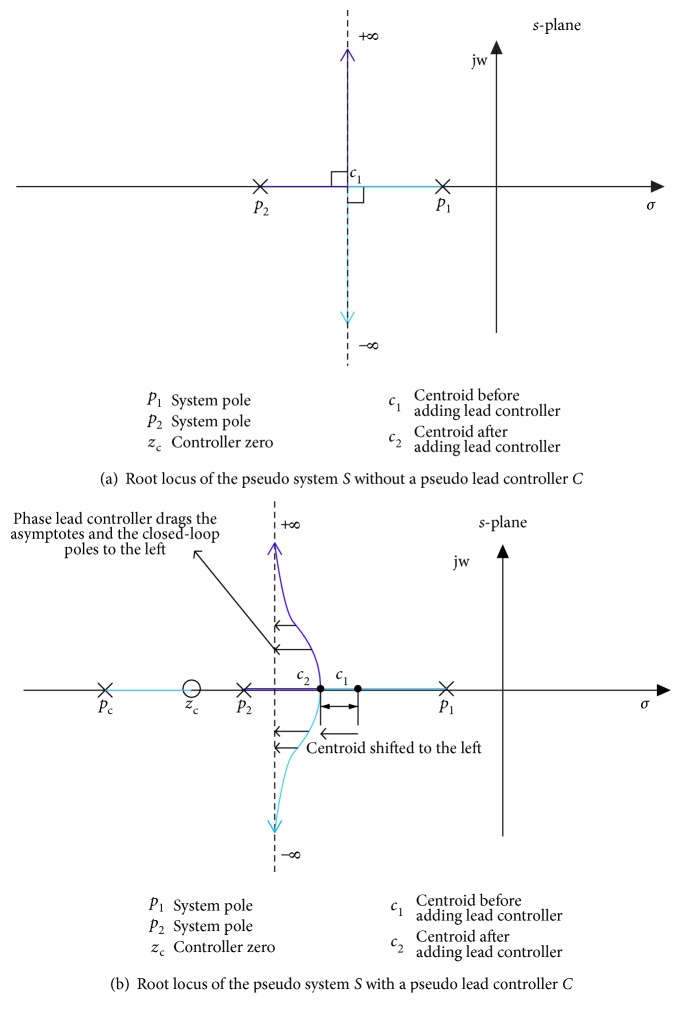
Graphical illustration of effects of a phase-lead controller on root locus using a pseudo system *S* and a pseudo lead controller *C*.

To achieve a faster response time, a phase-lead controller is designed, using the root locus method. In a root locus plot, the location where the asymptotes cross the real line is called the center of gravity or the centroid, defined in (15). A lead controller shifts the root locus towards the left side of the *s*-plane by shifting the centroid, the asymptotes, and the closed-loop poles to the left, thus providing a faster response [36]. Figures 7(a) and 7(b) graphically illustrates the effects of a phase-lead controller in terms of centroid movement and root locus shaping by considering a second-order pseudo system *S* and a pseudo phase-lead controller *C*.

(25) $$\begin{array}{c} Centroid=\frac{\sum finite\;poles-\sum finite\;zeros}{n-m}, \end{array}$$

where *n* = number of poles and *m* = number of zeros.

Now, to achieve the desired output, the actual phase-lead controller is designed using the following steps:

1. The zero of the controller is kept near the imaginary axis, as compared to the pole of the controller.
2. The pole of the controller is kept far from both the zero of the controller and the imaginary axis.
3. The distance between the pole and the zero of the controller is kept large.
4. The added pole has a larger negative value than the added zero, as zero resides near the origin.

**Figure 8:**
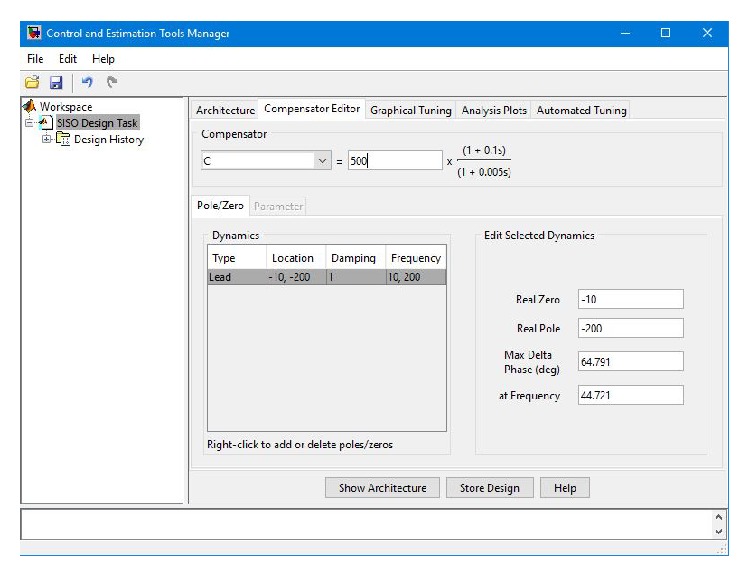
Illustration of the design details of the phase-lead controller.

The design details of the phase-lead controller are illustrated in Figure 8. The transfer function of the designed phase-lead controller with gain *k* = 500 is given as

(26) $$\begin{array}{c} G_{c}\left(s\right)=\frac{10,000\left(s+10\right)}{s+200}. \end{array}$$

The designed phase-lead controller and the dilution chamber in a closed-loop system, resulted in the following transfer function:

(27) $$\begin{array}{c} G_{CL}\left(s\right)=\frac{1000\left(s+10\right)}{\left(s+8.393\right)\left(s+1192\right)}. \end{array}$$

#### 3.2.4. Phase-Lead-Lag Controller

Both phase-lead and lag controllers have their own advantages and disadvantages as discussed above. Practical systems often demand certain rigorous specifications, where a combination of both lead and lag controllers can be practicable. The generalized transfer function of a phase-lead-lag controller is

(28) $$\begin{array}{c} C\left(s\right)=\frac{a_{1}\tau_{1}s+1}{\tau_{1}s+1}\cdot \frac{a_{2}\tau_{2}s+1}{\tau_{2}s+1}, \end{array}$$

where

(29) $$\begin{array}{c} a_{1}>1,a_{2}<1. \end{array}$$

To attain both faster response and relative stability, a phase-lead-lag controller is designed using the same design principles as have been discussed in the phase-lead and phase-lag sections. The following two steps describe the whole design process.

In the first step, a phase-lead controller is designed using the following procedure:

1. The pole of the controller is placed farther into the left half plane away from the imaginary axis.
2. The zero of the controller is placed near the imaginary axis.
3. The pole and zero of the controller are placed at such points, so that the distance between them is large enough and the centroid is shifted further to the left half plane.

In the second step, a phase-lag controller is designed with a different approach due to the succeeding reason. In a traditional lag controller design, the main target is to eliminate steady state error without varying the transients and root locus shaping. Instead of implementing the conventional approach, here, the main target is to achieve *both* elimination of steady state error as well as a fast response. For such requirement, varying the transients and root locus shaping is needed, which is accomplished in a different way, described as following:

1. Unlike the traditional pole zero placement of a lag controller, zero of the controller is placed away from both the pole of the controller and the imaginary axis.
2. Unlike in (18), *θ*_*P*_ − *θ*_*Z*_ is kept greater than 0, instead of closer to 0, where *θ_p_* and *θ_z_* are the angles associated with the pole and zero of the controller, respectively.

**Figure 9:**
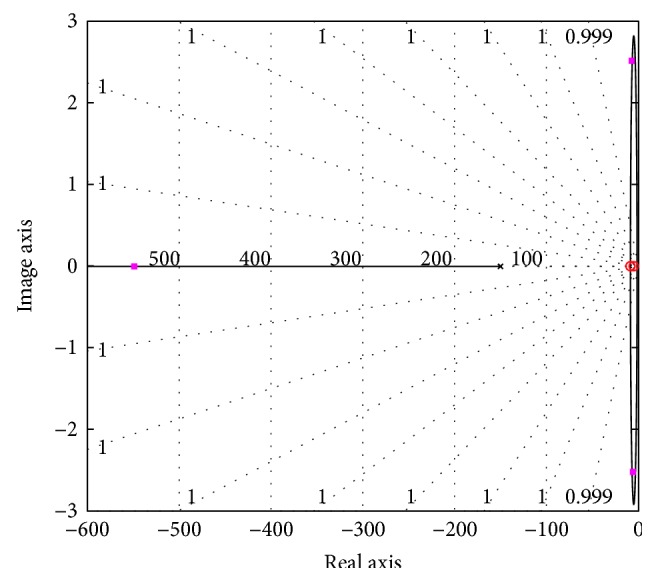
Root locus of the system incorporated with the designed lead lag controller.

A small amount of root locus shaping due to *θ*_*p*_ − *θ*_*z*_ ≠ 0 is verified and illustrated in Figure 9.

**Figure 10:**
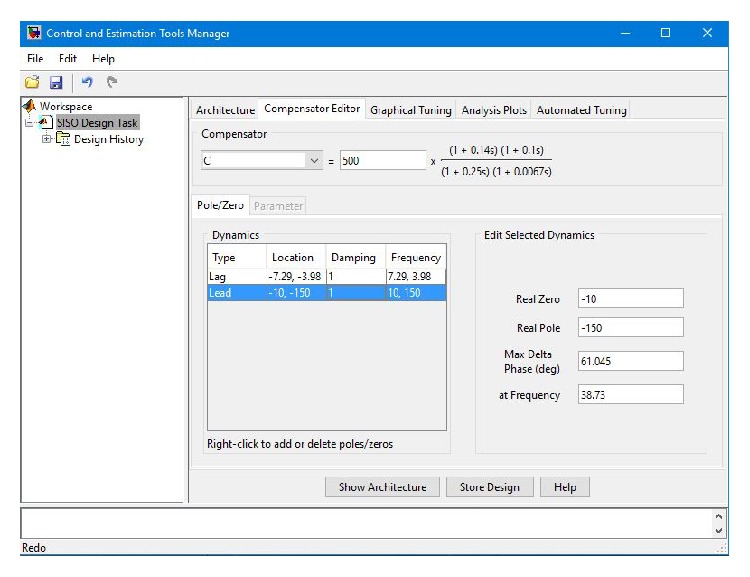
Illustration of the design details of the phase-lead lag controller.

The design details of the phase-lead-lag controller are illustrated in Figure 10. The transfer function of the designed lead-lag controller with gain, *k* = 500 results as follows:

(30) $$\begin{array}{c} G_{c}\left(s\right)=\frac{4094.7\left(s+7.29\right)\left(s+10\right)}{\left(s+3.98\right)\left(s+150\right)}. \end{array}$$

The controller when incorporated with the dilution chamber resulted in the following closed-loop transfer function:

(31) $$\begin{array}{c} G_{CL}\left(s\right)=\frac{409.47\left(s+7.29\right)\left(s+10\right)}{\left(s+549.6\right)\left(s^{2}+13.87s+54.32\right)}. \end{array}$$

#### 3.2.5. Cascaded Lead Controller

Generally, a cascaded phase-lead controller is applied to attain a very fast response time. Applying two lead controllers in series, merged with the plant in a closed-loop feedback system, can significantly accelerate the system response time. In case of a drug infusion system, a faster response is required because a small delay or overshoot in drug delivery can lead to severe consequences. The transfer function of a traditional cascaded lead controller is

(32) $$\begin{array}{c} C\left(s\right)=\frac{s+z_{1}}{s+p_{2}}\cdot \frac{s+z_{2}}{s+p_{2}}, \end{array}$$

where

(33) $$\begin{array}{c} p_{1}>z_{1},\;p_{2}>z_{2}. \end{array}$$

To achieve a much higher response time, a cascaded phase-lead controller is designed. The design principles are similar to what has been discussed in the phase-lead section. To achieve maximum desired output, consider the following:

1. Both poles of the two lead controllers are placed farther from the imaginary axis into the left half plane.
2. Both zeros of the two lead controllers are placed near the imaginary axis.
3. Maximum distance between the poles (*p*_1_, *p*_2_) and zeros (*z*_1_, *z*_2_) of the two lead controllers is kept.

Mathematically,

(34) $$\begin{array}{c} p_{1}-z_{1}\gg average\;distance,\\ p_{2}-z_{2}\gg average\;distance, \end{array}$$

where *p*_1_ and *p*_2_ represent the poles and *z*_1_ and *z*_2_ represent the zeros of the two cascaded lead controllers.

**Figure 11:**
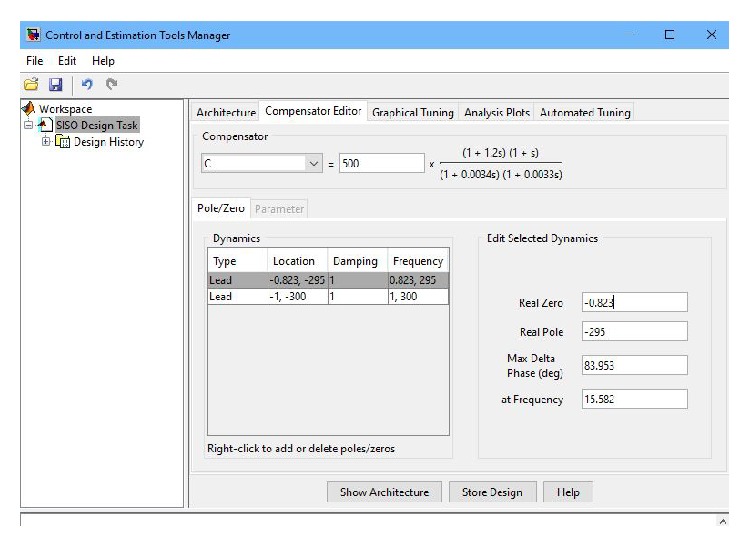
Illustration of the design details of the cascaded lead controller.

The design details of the cascaded lead controller are illustrated in Figure 11. The transfer function of the designed cascaded phase-lead controller with gain *k* = 500 is

(35) $$\begin{array}{c} G_{c}\left(s\right)=\frac{5.3767\times 10^{7}\left(s+0.823\right)\left(s+1\right)}{\left(s+295\right)\left(s+300\right)}. \end{array}$$

The designed cascaded phase-lead controller, when incorporated with the drug infusion system, resulted in the following closed-loop transfer function:

(36) $$\begin{array}{c} G_{CL}\left(s\right)=\frac{5.3767\times 10^{6}\left(s+1\right)\left(s+0.823\right)}{\left(s+5.377\times 10^{6}\right)\left(s+1.07\right)\left(s+0.7688\right)}. \end{array}$$

## 4. Results

### 4.1. Open-Loop Results

**Figure 12:**
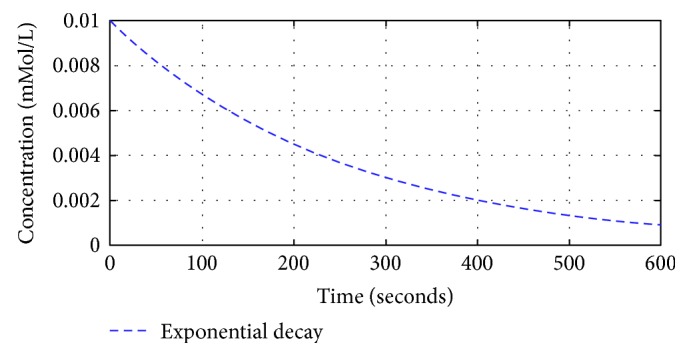
Exponential decay in concentration with propofol concentration = 0.01 mM and dilution flow rate = 0.04 mL/s.

To understand the first-order exponential decay of the dilution chamber, open-loop characteristics of the system were assessed in two steps in Section 2 of the open-loop analysis. The first step describes the dilution chamber in terms of its exponential decay property. The initial concentration of the propofol was set to 0.01 mMol/L in a fixed 10 mL volume of the dilution chamber. The result in Figure 12 shows and substantiates an exponential decay of concentration over time in the dilution chamber, due to the continuous dilution of the background electrolyte. The resultant plot is in perfect agreement with the theoretical computations using (4) and (5)

(37) $$\begin{array}{c} {\mathbb{C}}_{1}\left(t\right)=0.01e^{-0.004t}. \end{array}$$

In the second step, identification and validation of the open-loop gain and steady state analysis of the system is performed. Theoretically, the steady state gain is calculated from (7) in the system model and description. At steady-state *d*ℂ_1_(*t*)/*dt* goes to zero, and the open-loop gain *G* of the system will be

(38) $$\begin{array}{c} G=\frac{{\mathbb{C}}_{1}\left(t\right)}{i\left(t\right)}=\frac{1}{\lambda V_{1}}=\frac{1}{\Omega }=25\;s/mL. \end{array}$$

Propofol infusion, in parallel with the continuous dilution, is carried out for a certain duration. After some time, an equilibration state is achieved between the infusion and elimination rate of propofol. At such point, *d*ℂ_1_(*t*)/*dt* goes to zero and the concentration level approaches its steady state.

**Figure 13:**
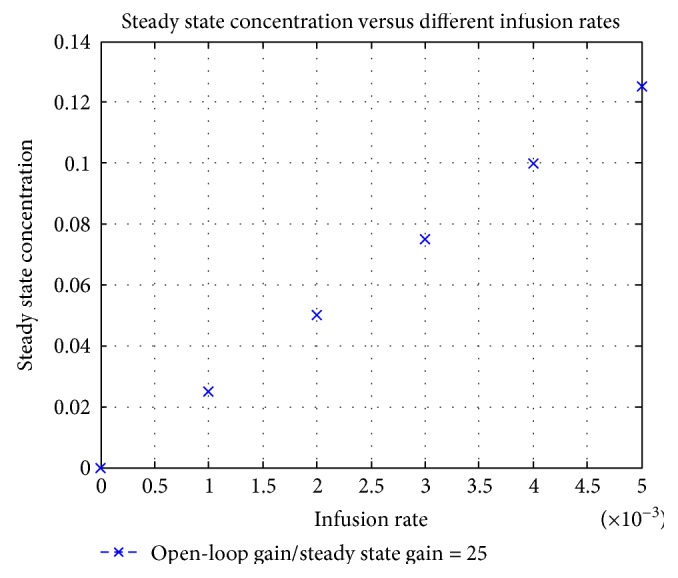
Steady state concentration at five different infusion rates with a flow rate, *Ω* = 0.04 mL/s.

The above infusion-dilution equilibrium led to the succeeding conception. For a particular value of dilution flow rate, a defined infusion rate results into a specific steady state concentration point. For a dilution flow rate of 0.04 mL/s, at five different infusion rates, the open-loop gain and the corresponding steady state concentration is evaluated and illustrated in Figure 13. The theoretical values calculated from (38) for different infusion rates completely meet the linear ratio as illustrated.

### 4.2. Closed-Loop Results

**Figure 14:**
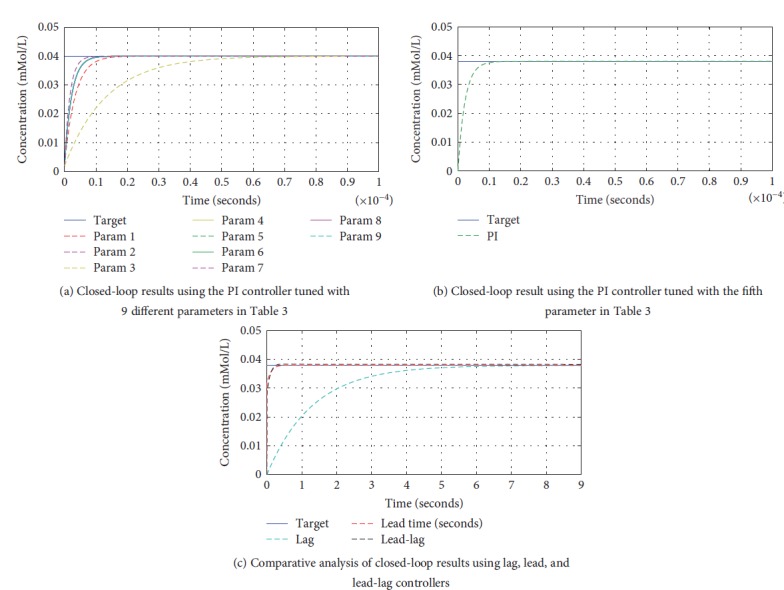
Comparative analysis of closed-loop results using PI, lead, lag, and lead-lag controllers.

Five different control schemes were employed in order to achieve the target drug concentration in a closed-loop feedback system. The PI controller was tuned with nine different parameters, shown in Table 3, with various parameters showing closer results to each other leading to overlapping response time, as shown in Figure 14(a). The effectiveness of *K_P_* and *K_I_* can be depicted from the graph in the following manner. The proportional term *K_P_* corresponds to the high rise time and settling time while the integral term *K_I_* corresponds to an improved final value of the response. The PI controller, when tuned with the fifth parameter in Table 3, yields a more effective response time in comparison with other nine parameters, as shown in Figure 14(b).

Figure 14(c) illustrates the comparison between a phase-lag, lead, and lead-lag controllers when incorporated with the dilution chamber in a closed loop. The phase-lag controller significantly improves the steady state error of the system, when compared with the open-loop step response in Figure 2: however, it does not contribute much to the response time as required. The phase-lead controller provides a much faster response as compared to a phase-lag controller as expected but still does not meet the higher response time requirements. The phase-lead-lag controller provides *both* faster response and steady state error reduction and therefore meets the objective as desired in the phase-lead-lag section of Section 3. The response time using a lead-lag controller seems closer to a single lead controller but better than a single lag controller.

**Figure 15:**
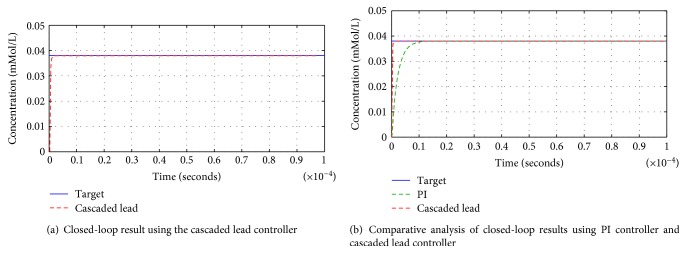
Comparative analysis of closed-loop results using cascaded lead controller and PI controller.

Figure 15(a) shows the response time of the system when incorporated with a cascaded lead controller in a closed-loop feedback system. Due to two lead controllers in series and effective pole zero placement, the response time is much faster as compared to all the other control techniques discussed above.

Figure 15(b) shows the comparative analysis between the closed-loop results using PI and cascaded lead controller. The PI controller and cascaded lead controller both result into a much faster and stable response as compared to other controllers; however, the cascaded lead controller shows relatively better results than the PI controller.  Table 4 scrutinizes the pros and cons of all five controllers in terms of the different time domain characteristics for each control technique.

## 5. Discussion

To target and maintain the drug concentration level in the human body, represented by a dilution chamber, five different control schemes are designed and implemented with a simulation-based comparative analysis between each of them. The comparative simulation study elucidates the effectiveness of each controller in targeting and maintaining the propofol level.

The drug concentration and dynamics are represented using the constant volume dilution chamber, due to the succeeding two *phenomena*. These are exponential decay of drug concentration in the dilution chamber and the steady state analysis of the system, explained thoroughly in step 1 and step 2 of the open-loop analysis, in Section 3. The open-loop results in Section 4 also gives a concrete substantiation of both *phenomena.*

In our *simulation-based study*, the results shown using a PI controller incorporated with the dilution chamber demonstrate a fast response time with a negligible amount of overshoot and no steady state error percentage. However, the results generated by Myers et al., using the same PI controller, showed a fast response time with a high overshoot, little steady state error, and some RMSE target percentage. These findings were due to the real-time analysis and an electrochemical sensor for feedback measurement. Following the PI controller, phase-lag, phase-lead, and phase-lead-lag controllers and a cascaded lead controller were designed and tuned using the root locus method in order to meet the desired requirements. Each controller shows its own merits and demerits in terms of different time domain specifications as shown in Table 4. The gain *k* is the same for all controllers, which is *k* = 500 except for the PI controller and phase-lag controller. For the PI controller, a much higher gain of *K_P_* = 4.5 × 10^6^ is used, making it more vulnerable to noise, when used in real-time analysis. For the phase-lag controller, a gain of *k* = 30 is used, which is essential in order to meet the desired steady state requirement. By using a cascaded lead controller, due to the increased centroid movement, the closed-loop poles of the system move further to the left half plane in the Laplace domain, providing a more quicker response as required. The cascaded lead controller surpasses all the other control techniques, most significantly with a lower gain value of *k* = 500. By employing such technique, the cascaded lead controller with a lower gain value provides relatively better results and response time than the PI controller with a much higher gain value, asserting cascaded lead controller as the most consummate technique.

The comparative analysis of the closed-loop results along with the time domain characteristics illustrated in Table 4 evidently demonstrates and validates the enhanced efficacy of a cascaded lead controller in a closed-loop drug infusion system.

## 6. Conclusion

Targeting and maintaining the drug concentration level are indispensable in human anatomy and hold central importance as an application of control technology in biomedical engineering. For a closed-loop drug infusion system, simulation of five different controllers has been demonstrated using MATLAB, which shows encouraging results for the delivery of propofol. For faster and stable response of the drug infusion system, comparative analysis for abovementioned controllers has been performed and analyzed. Following the simulation analysis and the results obtained, the cascaded lead controller and PI controller show the finest results with the cascaded lead controller showing relatively better results than all the other control techniques. The assertive results provide a key platform to implement this model in real time for automatic drug delivery. Such quintessential paradigm in the domain of biomedical control jargon would lead to faster response induction and reduction of clinical workload in the field of total intravenous anesthesia.

## Figures and Tables

**Table 1:** Simulation parameters.

Variable	Description	Value
*i*(*t*)	Infusion rate	Controller calculated infusion rate
ℂ_1_(*t*)	Concentration of the dilution chamber	Time dependent
Target	Target concentration of the dilution chamber	0.038 mMol
*𝕍*_1_	Volume of the dilution chamber	10 mL
*Ω*	Speed of dilution pump/dilution flow rate	0.04 mL/s
*λ*	Elimination rate *λ* = *Ω*/*𝕍*_1_	0.004 *s*^−1^

**Table 2:** Effects of *K_P_*, *K_D_*, and *K_I_* in a PID controller.

Response	Rise time	Overshoot	Settling time	Steady state error
*K_P_*	Decrease	Increase	No effect	Decrease
*K_I_*	Decrease	Increase	Increase	Eliminate
*K_D_*	No effect	Decrease	Decrease	No effect

**Table 3:** PI parameters.

PI parameters	*K_P_*	*K_I_*
1	3,000,000	55
2	6,000,000	55
3	750,000	55
4	4,500,000	5.5
5	4,500,000	55
6	4,500,000	550
7	4,500,000	5500
8	4,500,000	55,000
9	4,500,000	550,000

**Table 4:** Controller performance characteristics (a comparative analysis).

Controller	Gain	Rise time (sec)	Settling time (sec)	Overshoot (%)
Lag	30	2.9120	5.2720	0
Lead	500	0.0573	0.2492	0
Lead-lag	500	0.0880	0.1980	0.7164
PI	4.5 × 10^6^	4.8822 × 10^−6^	8.6935 × 10^−6^	0
Cascaded lead	500	4.0862 × 10^−7^	7.2779 × 10^−7^	0
